# Supplementary material for: Burden and predictors of anxiety disorder among HIV patients on ART in Nairobi Kenya
Source: PLOS Ment Health. 2024 Jul 1;1(2):e0000072. doi: 10.1371/journal.pmen.0000072 (PMC12798568; doi:10.1371/journal.pmen.0000072)
Supplement: Attachment — Submitted filename: PLOSOne_Human_Subjects_Research_Checklist.docx [file pmen.0000072.s003.docx]

**Human Participants Research Checklist**

***Complete the following if your study involved human participants or human participants’ data. These questions should be addressed for prospective and retrospective studies.***

1. Did you obtain ethics approval for this study? **YES,** this study was approved by the University of Nairobi / Kenyatta National Hospital Ethical Review Committee (KNH-ERC/A/413) and research license granted by the **National Commission for Science and Technology (NACOSTI)** (No: NACOSTI/P/22/21476. Before recruitment in this study, all patients filled in a written informed consent for study participation.
   - If yes, please upload (file type “Other”) the original approval document you received from your ethics committee. If the original document is in another language, please also provide an English translation.

**Uploaded**

- If you did not obtain ethical approval, please explain why this was not required below.

2. If you prospectively recruited human participants for the study – for example, you conducted a clinical trial, distributed questionnaires, or obtained tissues, data or samples for the purposes of this study, please report in the Methods:
   i. the day, month and year of the **start and end** of the recruitment period for this study. **The study was conducted between 1^st^ February, 2023 and 31^st^ May, 2023. As stated in the method section (Page 4).**
   ii. whether participants provided informed consent, and if so, what type was obtained (for instance, written or verbal, and if verbal, how it was documented and witnessed). If your study included minors, state whether you obtained consent from parents or guardians. If the need for consent was waived by the ethics committee, please include this information.

**Written informed consent was obtained from each and every participant recruited in the study (page 4)**

**Completed**

3. If you are reporting a retrospective study of medical records or archived samples, please report in the Methods section: **This was not a retrospective study**
i. the day, month and year when the data were accessed for research purposes
ii. whether authors had access to information that could identify individual participants during or after data collection

**N/A**
