# Supplementary material for: Burden and predictors of anxiety disorder among HIV patients on ART in Nairobi Kenya
Source: PLOS Ment Health. 2024 Jul 1;1(2):e0000072. doi: 10.1371/journal.pmen.0000072 (PMC12798568; doi:10.1371/journal.pmen.0000072)
Supplement: Attachment — Submitted filename: 'Response to Reviewers'.docx [file pmen.0000072.s004.docx]

Matteo Monzio Compagnoni, Ph.D.
Academic Editor

PLOS Mental Health

**RE: Response to reviewers’ comments for the manuscript PMEN-D-23-00061
Anxiety disorder and predictors among HIV patients in Nairobi Kenya
PLOS Mental Health**

Find a point by point responses to the issued raised by editor and reviewer(s).

1. Please amend your detailed online Financial Disclosure statement. This is published with the article. It must therefore be completed in full sentences and contain the exact wording you wish to be published.

**Response: the Financial Disclosure statement has been rewritten in full sentences**

a) State the initials, alongside each funding source, of each author to receive each grant. For example: &quot;This work was supported by the National Institutes of Health (####### to AM; ###### to CJ) and the National Science Foundation (###### to AM).&quot;
b) State what role the funders took in the study. If the funders had no role in your study, please state: &ldquo;The funders had no role in study design, data collection and analysis, decision to publish, or preparation of the manuscript.&rdquo;

**Response: The initials of each author and funding source have been inserted. The role of funders has also been clarified. As follows: KKG; Research work was funded by Health-Professional Education Partnership Initiative (HEPI)-Kenya a National Institute of health (NIH) Funded Project-R25TW011212. The funders had no role in study design, data collection and analysis, decision to publish, or preparation of the manuscript.**

2. Please update your online Competing Interests statement. If you have no competing interests to declare, please state: &ldquo;The authors have declared that no competing interests exist.&rdquo;

**Response: The online competing interest has been updated to read; The authors have declared that no competing interests exist**

3. In the online submission form, you indicated that &quot;Data will be provided by the corresponding author upon request&quot;.

All PLOS journals now require all data underlying the findings described in their manuscript to be freely available to other researchers, either

a) In a public repository,

b) Within the manuscript itself, or

c) Uploaded as supplementary information.

**Response: The data has been uploaded as supplementary information**

This policy applies to all data except where public deposition would breach compliance with the protocol approved by your research ethics board. If your data cannot be made publicly available for ethical or legal reasons (e.g., public availability would compromise patient privacy), please explain your reasons by return email and your exemption request will be escalated to the editor for approval. Your exemption request will be handled independently and will not hold up the peer review process, but will need to be resolved should your manuscript be accepted for publication. One of the Editorial team will then be in touch if there are any issues.

4. Please provide separate figure files in .tif or .eps format only and remove any figures embedded in your manuscript file. Please also ensure that all files are under our size limit of 10MB. You may leave the figure captions or legends in the manuscript.

**Response: The figure has been provided separately in tif or eps format and removed from the manuscript file. The figure file captions or legends have been provided in the manuscript**

For more information about how to convert your figure files please see our guidelines: https://journals.plos.org/mentalhealth/s/figures

5. We do not publish any copyright or trademark symbols that usually accompany proprietary names, eg (R), (C), or TM (e.g. next to drug or reagent names). Please remove all instances of trademark/copyright symbols throughout the text, including &reg; on pages 2 and 5.

**Response: copyright or trademark** (R) **in pages 2 and 5 have been remove**

6. We have noticed that you have uploaded Supporting Information files, but you have not included a list of legends. Please add a full list of legends for your Supporting Information files after the references list.

**Response: List of legends have been provided on page 19 for both the study data and the STROBE checklist**

Additional Editor Comments (if provided):

I&rsquo;ve read the work titled &ldquo;Anxiety disorder and predictors among HIV patients in Nairobi Kenya&rdquo; with pleasure. The paper is well written, quite simple but the results and the topic are quite interesting and actual, since they are referring to some of the more important neglected diseases in these last three decades: HIV and mental disorders (anxiety in this specific case).

Despite that, according also with the reviews received, which could be very usefule to improve the manuscript if well answered, I propose some major revisions. Following please you can find some brief suggestions by my side.

- The title it could be clearer. I guess it is slightly confusing. I mean, it is not clear what is being referred to. For example, it is not clear (from the title itself) whether the Authors studied predictors for HIV or for anxiety disorder. Furthermore, from the title and the abstract should emerge more clearly that the target population are patients living with HVI on treatment with ART.

**Response: This study provide data on anxiety disorder among HIV patients. This has been made clearer in the title and the abstract: The title has been changed to read Proportions and Predictors of Anxiety disorder among HIV patients on ART in Nairobi Kenya.**

**The first sentence of the abstract rewritten to read; anxiety disorders are prevalent among people living with Human Immunodeficiency Virus (HIV) globally, but data on the prevalence and factors associated with this mental illness are limited among HIV patients on ART in Kenya. The words among HIV have been inserted in line 23; this states the study population right away in the abstract.**

- Lines 57-58: mental health is really a human right, as stated in the WHO definition of health, but also mental health is neglected and still need more attention and interest in the public health context. Please modify this sentence.

**Response: The sentence has been modified as follows: Although mental health is a universal human right according to World Health Organization (WHO), the mental health of PLHIV is often neglected and still need more attention and interest in the public health context [11]. In low‐ and middle‐income countries (LMICs), mental health among PLHIV is only beginning to receive the deserving attention [12]. Lines 63-66**

- Line 61: please replace the term determine;. Maybe something like to assess/estimate the prevalence of anxiety among PLHIV receiving treatment at Mbagathi Hospital in Nairobi County. Furthermore, as a secondary aim, we evaluated predictors correlated with anxiety Or something like that. It should be clear that the Authors aimed at evaluating the prevalence of anxiety disorder and its predictors.

**Response: in line 68 –71 the sentence has been changed to read as follows: To help bridge this research gap in Kenya, we conducted a cross sectional study to estimated the prevalence among PLHIV receiving treatment at Mbagathi Hospital in Nairobi County. Furthermore, as a secondary aim, we evaluated predictors correlated with anxiety among this population.**

- Lines 71-72: the inclusion and exclusion criteria are quite clear. I guess that Authors should explain why patients receiving mental healthcare within or outside Mbagathi Hospital were excluded from the study.

**Response: Patients with mental conditions were excluded to minimize bias beside they were deemed unable to provide informed consent. As stated in line 82-84. Exclusion criteria included those receiving mental healthcare within or outside Mbagathi Hospital (to minimize bias and they were deemed unable to provide informed conset) and those unable to provide consent.**

- I guess the Authors should give a better description of the catchment area from which they selected the cohort study. I believe that a better description of the Mbagathi Hospitals; Comprehensive Care Clinic, with a description of how many patients are treated there, how many patients are assisted in general by this clinic, could give more interest and support a better comprehension.

**Response: Line 80 to 90. A description of Mbagathi County Hospital in the CCC clinic has been added.**

- Line 103: with anxiety; maybe could be replaced with; with the presence of symptoms related with moderate to severe anxiety

**Response: with anxiety has been replaced with the presence of symptoms related with moderate to severe anxiety: Lane 126**

- Please provide also mean and SD of descriptive statistics for quantitative variables. Or median, where more suitable.

**Response: The median (IQR) has been provided where appropriate (Table 1)**

- Line 117: identified, 43(21%) had;

- Lines 116-119: please use also the cutoffs of BAI in this sentence

**Response: Line 144 – 147: The cutoffs of BAI has been inserted as follows: The proportion of participants with a BAI score of 8 or more indicated moderate to severe anxiety. Using this categorization; there were 60 (29.3%) of the participants who were considered anxious (including mild, moderate and severe anxiety levels) compared to 145 (70.7%) of the participants considered not anxious**

- In table 3 insert in the header the total of patients in the group with presence of anxiety (60 I guess) and without the presence of anxiety (the other 145 patients, if I am not wrong).

**Response: Table 3 presence of anxiety (n = 60) and without the presence of anxiety (n = 145) inserted line 391**

- Please control and make sure for having defined all the acronyms and abbreviations along the manuscript (sSA, TB, etc.).

**Response all acronyms and abbreviations either removed or defined**

[Note: HTML markup is below. Please do not edit.]

Reviewers; comments:

Reviewers Responses to Questions

**Comments to the Author**

1. Does this manuscript meet PLOS Mental Health&rsquo;s [publication criteria](https://journals.plos.org/mentalhealth/s/criteria-for-publication)? Is the manuscript technically sound, and do the data support the conclusions? The manuscript must describe methodologically and ethically rigorous research with conclusions that are appropriately drawn based on the data presented.

Reviewer #1: Yes

Reviewer #2: Partly

2. Has the statistical analysis been performed appropriately and rigorously?

Reviewer #1: Yes

Reviewer #2: No

3. Have the authors made all data underlying the findings in their manuscript fully available (please refer to the Data Availability Statement at the start of the manuscript PDF file)?

The [PLOS Data policy](https://journals.plos.org/mentalhealth/s/data-availability) requires authors to make all data underlying the findings described in their manuscript fully available without restriction, with rare exception. The data should be provided as part of the manuscript or its supporting information, or deposited to a public repository. For example, in addition to summary statistics, the data points behind means, medians and variance measures should be available. If there are restrictions on publicly sharing data&mdash;e.g. participant privacy or use of data from a third party&mdash;those must be specified.

Reviewer #1: Yes

Reviewer #2: Yes

4. Is the manuscript presented in an intelligible fashion and written in standard English?

PLOS Mental Health does not copyedit accepted manuscripts, so the language in submitted articles must be clear, correct, and unambiguous. Any typographical or grammatical errors should be corrected at revision, so please note any specific errors here.

Reviewer #1: Yes

Reviewer #2: No

5. Review Comments to the Author

Please use the space provided to explain your answers to the questions above. You may also include additional comments for the author, including concerns about dual publication, research ethics, or publication ethics. (Please upload your review as an attachment if it exceeds 20,000 characters)

Reviewer #1: I have read with interest the work titled;Anxiety disorder and predictors among HIV patients in Nairobi Kenya.

The paper is original and of high interest to readers. Moreover, the objective of the paper is really interesting and actual, and this manuscript could provide a useful contribution to the literature.

I propose some minor revisions.

By section:

Introduction

1. The introduction is well written and summarizes the current state of the art well. The rationale is clear and the literature used is recent. In line 55, I would add the references to give greater support to the statement.

**Response: Additional references added including Spies, et al.. AIDS Behav., 22(10), 3155–65 and Cavazos‐Rehg et al., AIDS Behav., 24(9), 2546–54.**

Materials and Methods

2. The materials and methods are generally well written. Nevertheless, I would add more details to some passages in order to make this part more informative. What is the source of the data, for example? When were they collected? Specifying the sources of the data enables you to understand a whole series of biases (e.g., selection bias and information bias). In studies such as this one that evaluate prevalence, representativeness is essential. Cross-sectional studies of diseases, traits, or other problems are therefore susceptible to selection bias. In cross-sectional studies as in any other, information bias, the inaccurate measurement or recording of a disease or characteristic, is also a key issue.

**Response: The following additional information has been added**

1. **When the data was collected has been provided in line 78: 1^st^ February, 2023 and 31^st^ May, 2023 among People Living with HIV (PLHIV) receiving antiretroviral treatment (ART) at Mbagathi Hospital, Nairobi County**
2. **Additional information about the study site, the number of HIV patients seen has been added: Line 80 to 90**
3. **The actual process of data collection has been added in page 5; line 110 to 118**

3. In line 67 it is reported; This cross-sectional study was conducted between 1st February, 2023 and 31st May, 2023;. Can you explain why this date range was chosen? Is it related to the studys rationale or to data availability? It would be beneficial to add more details to the study.

**Response: The dates between 1st February, 2023 and 31st May, 2023 are the actual dates the study was conducted. We anticipate that this will point the reader as to when the study was done**

4. According to line 69; Eligible participants had a documented HIV diagnosis; what do you mean by; documented HIV diagnosis;? Whenever possible, it is best to report a pathology identification code. Also, when was it verified? This information is always good to provide, even if it may seem obvious. Regarding the cohort selection, I would like to add more details (also supported by the supplementary materials).

**Response: the sentence changed to read Eligible participants were those that tested HIV positive according to the Kenyan guideline and were enrolled into HIV care and treatment program**
5. What is the purpose of including patients aged; 18 years and excluding subjects aged 18 years?

**Response: The question is not very clear, however, the study enrolled those of consenting age ≥ 18 years; the symbol greater than or equal to have been inserted. Line 92**

6. The exclusion criteria are reported in lines 71-72. Also in this case, did you limit yourself to excluding the subjects who received assistance in the period 1st February, 2023 and 31st May, 2023? Or did you also exclude those who had also received assistance in previous periods (for example in the previous 5 years)?

**Response: Those excluded were within the study period**

Despite the studys design, it is advisable to include these details anyway to avoid any misunderstandings on the part of the reader.

**Response: More detail about the study design, methodologies and enrollment strategies have been provided in line 80 to 90 and Line 131 to 139**

7. What do you mean by; those receiving mental healthcare;? Please specify all the services considered, the reasons why they were excluded, the period and any service identification codes (also, in this case, I would suggest adding specifications in the supplementary materials).

**Response: The section has been revised for clarity. The phrase receiving mental healthcare was meant to show the patient who had underling mental conditions were excluded because they were deemed unable to give informed consent. The sentence has since been deleted**

8. Line 72. What do you mean by those unable to provide consent;? Adding more details would be appropriate in this case. Having a more accurate definition of these subjects allows us to understand the potential nonresponse bias. That is, any systematic difference between respondents (people who complete a survey) and non-respondents (people who do not complete a survey).To make the study generalizable and above all replicable, you should add more details in this paragraph.

**Response: What we meant here is that as stated in the methods section, this study used systematic random sampling to invite every third patient attending the HIV clinic during the study period (These patients were generated for existing database in the clinic). Not all accepted to participant and were unable to provide consent. This is what we meant by this statement**

9. Lines 89-97: add more details regarding the procedure through which the data were collected.
**Response: Additional details on data gathering has been provided in the Data collection Instruments. Lines 131 to 139**

10. Lines 99-100. In this paragraph, it would be useful to indicate which descriptive statistics were used. It would also be useful to add the standard deviation to those you entered.
**Response: The data analysis section has been revised to capture all aspect of data analysis as copied herein. Data analysis was performed using R software version 4.1.2. Descriptive statistics summarized the sociodemographic, socioeconomic, and clinical data. The median and interquartile range (IQR), was used to report two numeric variables (Age and Household Size) to minimize the effects of outliers and skewness in the data. The frequency counts and percentages were used to express all categorical variables. The proportion of participants with a BAI score of 8 or more, indicating moderate to severe anxiety, was calculated. Bivariable logistic regression analyses assessed the association of each variable (demographic and clinical) with the presence of symptoms related with moderate to severe anxiety. A multivariable logistic regression model explored the independent associations between identified factors and anxiety levels. The variables included in the multivariate logistic regression model were purposively selected as described by Lemashow et al. [14]. The variables that were first significant with a Wald test were selected. All the variables that were significant at a cut-off p-value of 0.2 were included in the model to enable identification of all important variables in the study. The significance level was set at P ≤ 0.05.**

11. Have you used some specific procedure for the selection of the variables that were used for the estimation of the multivariate logistic regression model (i.e., backward elimination with the Akaike information criterion or modern shrinkage or penalization procedures, such as LASSO/least absolute shrinkage and selection operator, elastic net, and their variants.)?

**Response: The section addressing multivariate has also been provided as copied herein: A multivariable logistic regression model explored the independent associations between identified factors and anxiety levels. The variables included in the multivariate logistic regression model were purposively selected as described by Lemashow et al. [14]. The variables that were first significant with a Wald test were selected. All the variables that were significant at a cut-off p-value of 0.2 were included in the model to enable identification of all important variables in the study.**

Results
12. In general, I would always add the same number of decimals when reporting results (e.g., typo in line 113).

**Response: The decimal mismatched has been corrected line 169: Viral suppression (less than 50 copies/mL) was observed in 168 (81.9%) of the participants, and 191 (93.2%)**

13. In order to simplify reading for the reader, the numbers (with any approximations) must be the same in the tables. It is recommended that you correct any typos in the following lines: (i) 124 (p = 0.027, the table shows p = 0.026); (ii) 130 (p = 0.0104, the table shows p t; 0.001); (iii) 132 (p = 0.0125, the table shows p = 0.013).

**Response: The following typos have been corrected**

(i) **(p = 0.027, changed to p = 0.026) Line 18**
(ii) **(p = 0.0104, changed to p < 0.001) Line 193**
(iii) **(p = 0.0125 changed to p = 0.013) Line 195**

Reviewer #2: This study aimed to investigate the prevalence and correlates of anxiety disorder among HIV patients receiving care and treatment at a comprehensive care clinic in Nairobi, Kenya. However, several issues need to be addressed:

-The authors did not provide an explanation for why mental health issues among People Living with HIV (PLHIV) in low‐ and middle‐income countries (LMICs) should receive deserving attention. The introduction section lacks detail and fails to contextualize the significance of mental health concerns in this population within the broader socioeconomic and healthcare landscape of LMICs.

**Response: Significance of Mental Health Concerns in LMICs: In the introduction first paragraph shows the importance of anxiety symptoms among PLHIV. The sentence further shows PLHIV are twice as likely as the general population to be diagnosed with an anxiety disorder (mental disorders) [2]. The second paragraph then show the magnitude of anxiety in developing nations but also highlight the significant lack of data in LMICS justifying why this and similar studies among LMICS are important and urgently needed. The last paragraph further stresses the lack of data setting the basis for this study.**

**In low‐ and middle‐income countries, mental health among PLHIV is only beginning to receive the deserving attention [14].**

-The study did not specify the inclusion or exclusion criteria for participant selection. Clear criteria are essential for ensuring the sample is representativeness and the study is generalizability.
**Response: The inclusion and exclusion criteria were clearly defined in our methods section; however, we appreciate that further clarification could enhance the manuscript. We have included additional details on the process of participant selection to ensure our sample's representativeness and the generalizability of the study findings.**

- Depression and anxiety often coexist and can have overlapping symptoms. However, the study did not include screening for depression, which is a common comorbidity among individuals with anxiety disorders. Failure to assess depression limits the comprehensive understanding of mental health issues among the study participants and may lead to incomplete conclusions.

**Response: Screening for Depression: Response: Your observation is indeed pertinent. Depression and anxiety are often comorbid, and their symptoms can overlap, impacting the accuracy of anxiety assessments. While our study focused on anxiety, we acknowledge that assessing for depression could provide a more comprehensive understanding of mental health in our cohort. We propose to discuss this limitation and suggest the need for integrated screening for both conditions in future research within our discussion section.**

6. PLOS authors have the option to publish the peer review history of their article ([what does this mean?](https://journals.plos.org/mentalthealth/s/editorial-and-peer-review-process#loc-peer-review-history)). If published, this will include your full peer review and any attached files.


**Do you want your identity to be public for this peer review?** If you choose &ldquo;no&rdquo;, your identity will remain anonymous but your review may still be made public.


For information about this choice, including consent withdrawal, please see our [Privacy Policy](https://www.plos.org/privacy-policy).

Reviewer #1: No

Reviewer #2: No

[NOTE: If reviewer comments were submitted as an attachment file, they will be attached to this email and accessible via the submission site. Please log into your account, locate the manuscript record, and check for the action link &quot;View Attachments&quot;. If this link does not appear, there are no attachment files.]

While revising your submission, please upload your figure files to the Preflight Analysis and Conversion Engine (PACE) digital diagnostic tool, https://pacev2.apexcovantage.com/. PACE helps ensure that figures meet PLOS requirements. To use PACE, you must first register as a user. Registration is free. Then, login and navigate to the UPLOAD tab, where you will find detailed instructions on how to use the tool. If you encounter any issues or have any questions when using PACE, please email PLOS at figures@plos.org. Please note that Supporting Information files do not need this step.

Thank you for your valuable comments which we have incorporated the suggested changes and we hope that manuscript meets the requirements of the journal.

Looking forward for a positive response from you

Kind regards,

Kevin Kibera Gacau

Department of Paediatrics and Child Health, University of Nairobi, Kenya.

Enclosed is our manuscript
