# Supplementary material for: Burden and predictors of anxiety disorder among HIV patients on ART in Nairobi Kenya
Source: PLOS Ment Health. 2024 Jul 1;1(2):e0000072. doi: 10.1371/journal.pmen.0000072 (PMC12798568; doi:10.1371/journal.pmen.0000072)
Supplement: Attachment — Submitted filename: Response to Reviewers.docx [file pmen.0000072.s005.docx]

PMEN-D-23-00061R1
Proportions and Predictors of anxiety disorder among HIV patients on ART in Nairobi Kenya
PLOS Mental Health

Dear Dr. Ngayo,

Thank you for submitting your manuscript to PLOS Mental Health. After careful consideration, we feel that it has merit but does not fully meet PLOS Mental Health’s publication criteria as it currently stands. Therefore, we invite you to submit a revised version of the manuscript that addresses the points raised during the review process.

Please submit your revised manuscript by **6th of May, 2024**. If you will need more time than this to complete your revisions, please reply to this message or contact the journal office at mentalhealth@plos.org. When you're ready to submit your revision, log on to https://www.editorialmanager.com/pmen/ and select the 'Submissions Needing Revision' folder to locate your manuscript file.

Please include the following items when submitting your revised manuscript:

- A rebuttal letter that responds to each point raised by the editor and reviewer(s). You should upload this letter as a separate file labeled 'Response to Reviewers'.
- A marked-up copy of your manuscript that highlights changes made to the original version. You should upload this as a separate file labeled 'Revised Manuscript with Track Changes'.
- An unmarked version of your revised paper without tracked changes. You should upload this as a separate file labeled 'Manuscript'.

Guidelines for resubmitting your figure files are available below the reviewer comments at the end of this letter.

We look forward to receiving your revised manuscript.

Kind regards,

Matteo Monzio Compagnoni, Ph.D.
Academic Editor
PLOS Mental Health

Journal Requirements:

1. We have noticed that you have a list of Supporting Information legends in your manuscript. However, there are missing files in the submission. Please upload them as separate files with the item type 'Supporting Information'. Or if the Supporting Information is no longer to be included as part of the submission please remove all reference to it within the text.

**Response: The study data has been uploaded as Supporting Information 1. Other supplementary documents, such as the STROBE checklist and study ethical approval, have been removed from the manuscript.**

2. Please review your reference list to ensure that it is complete and correct. If you have cited papers that have been retracted, please include the rationale for doing so in the manuscript text, or remove these references and replace them with relevant current references. Any changes to the reference list should be mentioned in the rebuttal letter that accompanies your revised manuscript. If you need to cite a retracted article, indicate the article’s retracted status in the References list and also include a citation and full reference for the retraction notice.

**Response: The references in the manuscript have been thoroughly reviewed and verified for accuracy. Any retracted papers have been replaced with current relevant references, and changes have been noted in the rebuttal letter**

Additional Editor Comments (if provided):

I’ve read the revised version of the work titled “Proportions and Predictors of anxiety disorder among HIV patients on ART in Nairobi, Kenya” (manuscript ID PMEN-D-23-00061-R1), by Musa Otieno Ngayo, et al, with pleasure. It is a simple, good study giving an insight on the burden of anxiety disorder HIV patients treated with antiretroviral therapy in a district of Kenya. I am pleased that the Authors have addressed all the comments raised by the Reviewers in the first round of peer-review. The paper is clearly improved, and I believe that it can contribute to the literature on HIV and anxiety, specifically in Africa.
However, according also with the suggestions received from the Reviewers, I believe that some MINOR REVISIONS should be made, before being considered completely fit for the publication standards of this Journal. Indeed, after having addressed these (minor) comments, the paper should be considered as worthy of publication.

Here some minor comments (to be considered in addition to those of the Reviewers):
- Line 25: Insert in the abstract the cut-off considered for the BAI score for a patient to be considered with anxiety symptoms.

**Response: The cut-off of BAI inserted as follows Inventory (BAI) Questionnaire was used to determine the prevalence of anxiety disorder with score of 8 or more, indicating moderate to severe anxiety**

- Line 25: replace “to gather attributes” with “to collect information on characteristics”.

**Response: gather attributes replaced with to collect information on characteristics as suggested**

- Line 27: please delete the information related to the software; it is not relevant for the abstract.

**Response: on R software version 4.1.2 deleted in line 27 as suggested**

- Line 34: replace “were more likely to have anxiety disorder” with “were factors associated with having anxiety disorder”.

**Response: The line replaced as suggested**

- Line 35: “Among PLHIV receiving ART in a cunty of Kenya, there is a considerable …”

**Response: The sentence redone to read as follows: Among PLHIV receiving ART in a county of Kenya, there is a considerable association of anxiety with sex, occupation, household population size and history of tuberculosis infection.**

- Line 47: delete “(mental disorder)”, is redundant.

- Line 47: replace “is bidirectional” with something less secure, like “seems to be bidirectional”.

**Response: Suggestion adopted**

- Line 49: Authors refers to “progression” of HIV, and what about the incidence?

**Response: The sentence qualifies the bidirectional relationship between anxiety disorder and HIV infection and not the rates or incidence of HIV. The line has been retained**
- Ine 51: please add a reference for the “29%”.

**Response: Reference inserted**

- Line 52: “prevalence of anxiety disorder among PLHIV…”.

**Response: Rewritten as suggested**

- Line 99: “… study, according to the inclusion criteria, all patients …”.

**Response: The sentence according to the inclusion criteria…. inserted into the line 106 as suggested**
- Line 102: delete “the researcher planned and organized” and use something like “The data collection process was planned and organized in accordance with the CCC in charge. The best time and day for questionnaire administration were identified to suite …”

**Response: The sentence rewritten as suggested page 5**

- Line 104: “were trained to administer”.

**Response: The sentence rewritten as suggested page 5**

- Line 107: “These research assistants gathered …”.

**Response: “The” deleted from the sentence as suggested page 5**

- Line 121: move “Data analysis was performed using R software version 4.1.2.” at the end of the Statistical Analysis section.

**Response: Done as suggested**

- Line 123: “was used for numeric variables”.

**Response: Done as suggested**

- Line 126: please insert a reference for the cutoffs used.

**Response: Reference added (16 and 17)**

- “bivariate” and “multivariate”, instead of “bivariable” and “multivariable”.

**Response: bivariate” and “multivariate used in the sentence**

- Please, check along the manuscript that is “Lemeshow” (reference #16) and not “LemAshow”.

**Response: Re-edited accordingly**

- Line 102: why authors used a cut-off p-value of 0.2? Please explain that or use a reference for that choice, instead of using 0.05, for example.

**Response: Previous research established that a cut-off p-value of 0.05 for variable selection is usually too stringent and leads to the exclusion of otherwise important variables. As a result, several studies have suggested a more ‘lenient’ range for variable selection (0.15 – 0.4). Following various simulations during the analysis, the cut-off p-value of 0.2 was settled upon as it best fit the data. This cut-off value was only used during variable selection for the variables to be included in the multivariate model. The assessments of significance during the univariate and multivariate testing, however, were all performed at a 0.05 level of significance.**

**Reference: Wang, Q., Koval, J. J., Mills, C. A., & Lee, K. I. D. (2007). Determination of the Selection Statistics and Best Significance Level in Backward Stepwise Logistic Regression. *Communications in Statistics - Simulation and Computation*, *37*(1), 62–72.** [**https://doi.org/10.1080/03610910701723625**](https://doi.org/10.1080/03610910701723625)

- Line 137: “A total of 205 participants with a median age of 49 years”.

**Response: Redone as suggested**

- Please, modify Table 1, in a more scientific template (like that adopted for Table 3, for example).

**Response: Table 1 is a descriptive table while table 3 has measures of association, the two cannot be similar**

- Line 150: “… of the participants considered not anxious, or without the presence of anxiety symptoms”.

**Response: Redone as suggested**

- In the Table 4, I suggest reporting only the results (aOR and 95% CI) only for the multivariate analysis, and not those obtained from the bivariate analysis. Reporting both it would be a bit redundant. Please, report these bivariate results in a supplementary table (exactly like Table 4, but supplementary).

**Response: Presenting both the bivariate and multivariate in one table eases the ability to confirm which variables were added into multivariate model. We request to leave the table as it is**

- Line 168: “anxiety, in a county of Kenya”, I suggest to not be too general and categorical, and to always report the geographical context.

**Response: Suggestion adopted**

- Line 190: “anxiety symptoms”.

**Response: The word symptoms inserted**

- Line 248: “The identified associated factors emphasize the multifaceted…”.
- I suggest to adopt a more frequent use of the impersonal third person in the sentence construction, all along the manuscript.

**Response: The sentence rewritten in third person language**

- I believe that a further proofread and language control should be made.

Response: The study proof read using the

[Note: HTML markup is below. Please do not edit.]

Reviewers' comments:

Reviewer's Responses to Questions

**Comments to the Author**

1. If the authors have adequately addressed your comments raised in a previous round of review and you feel that this manuscript is now acceptable for publication, you may indicate that here to bypass the “Comments to the Author” section, enter your conflict of interest statement in the “Confidential to Editor” section, and submit your "Accept" recommendation.

Reviewer #1: All comments have been addressed

Reviewer #3: All comments have been addressed

Reviewer #4: (No Response)

**Response: Thank you for this feedback**

2. Does this manuscript meet PLOS Mental Health’s [publication criteria](https://journals.plos.org/mentalhealth/s/criteria-for-publication)? Is the manuscript technically sound, and do the data support the conclusions? The manuscript must describe methodologically and ethically rigorous research with conclusions that are appropriately drawn based on the data presented.

Reviewer #1: Yes

Reviewer #3: Yes

Reviewer #4: Yes

**Response: Thank you for this feedback**

3. Has the statistical analysis been performed appropriately and rigorously?

Reviewer #1: Yes

Reviewer #3: Yes

Reviewer #4: Yes

**Response: Thank you for this feedback**

4. Have the authors made all data underlying the findings in their manuscript fully available (please refer to the Data Availability Statement at the start of the manuscript PDF file)?

The [PLOS Data policy](https://journals.plos.org/mentalhealth/s/data-availability) requires authors to make all data underlying the findings described in their manuscript fully available without restriction, with rare exception. The data should be provided as part of the manuscript or its supporting information, or deposited to a public repository. For example, in addition to summary statistics, the data points behind means, medians and variance measures should be available. If there are restrictions on publicly sharing data—e.g. participant privacy or use of data from a third party—those must be specified.

Reviewer #1: Yes

Reviewer #3: Yes

Reviewer #4: Yes

**Response: Thank you for this feedback**

5. Is the manuscript presented in an intelligible fashion and written in standard English?

PLOS Mental Health does not copyedit accepted manuscripts, so the language in submitted articles must be clear, correct, and unambiguous. Any typographical or grammatical errors should be corrected at revision, so please note any specific errors here.

Reviewer #1: Yes

Reviewer #3: Yes

Reviewer #4: Yes

**Response: Thank you for this feedback**

6. Review Comments to the Author

Please use the space provided to explain your answers to the questions above. You may also include additional comments for the author, including concerns about dual publication, research ethics, or publication ethics. (Please upload your review as an attachment if it exceeds 20,000 characters)

Reviewer #1: I have read the article now titled “Proportions and Predictors of Anxiety Disorder Among HIV Patients on ART in Nairobi Kenya”. I am pleased that the authors have addressed the issues raised in the previous round.

**Response: Thank you for this feedback**

Currently, the manuscript is a well-written research paper whose main purpose is to estimate the prevalence among PLHIV receiving treatment at Mbagathi Hospital in Nairobi, Kenya. While the secondary aim is to evaluate the predictors correlated with anxiety in the same population.

I believe the manuscript meets the publication standards of the journal.

**Response: Thank you for this feedback**

Reviewer #3: Proportions and Predictors of anxiety disorder among HIV patients on ART in Nairobi Kenya
General comments and some key concerns:
It is a good study giving an insight on the burden of anxiety disorder HIV patients on antiretroviral therapy (ART). However, below are my comments.
1. Title:
Line 1-2: Title need to be paraphrased as “Prevalence and Predictors of anxiety disorder among HIV patients on antiretroviral therapy (ART) in Nairobi Kenya” OR “Burden and Predictors of anxiety disorder among HIV patients on antiretroviral therapy (ART) in Nairobi Kenya”

**Response: Thank you for this comment. The title has been revised to read Burden and Predictors of anxiety disorder among HIV patients on antiretroviral therapy (ART) in Nairobi Kenya”**

Line 9: It should be written as:- Department of Pharmacy, Directorate of Clinical Services, Kiambu County, Nairobi, Kenya

**Response: the sentence edited as suggested**

2. Abstract
• Line 20: Replace “estimated” with “determined”

**Response: Replaced as suggested**
• Line 20: Sentence seems to be the aim but it doesn’t match with the title. It needs to be paraphrased

**Response:** The sentence in line 20 aligns with the main aim of the study and effectively conveys the message of the title using different words **Yes, the sentence is the main aim of the study which conveys the massage as the title but uses different words**

• Line 35: Seems to be a conclusion but it is missing the element of burden in it.

**Response: The conclusion has been revised to highlight the burden of anxiety: 'Among PLHIV receiving ART in Nairobi County, Kenya, there is a considerable burden of anxiety disorder associated with sex, occupation, household size, and history of tuberculosis infection.**

3. Introduction

• Line 65: prevalence of what??

**Response: The sentence has been clarified; prevalence of anxiety disorder**

4. Methodology
• Line 69: Materials and Methods should be replaced with “ Methodology”
• A number of important components of the methods are missing or mixed up and therefore not very clear
• Also, I suggest that the Methodology be sub-sectioned for ease of follow flow as below:
o Study design
o Study setting
o Study population
o Sample size
o Selection criteria – Inclusion and exclusion
o Sampling procedure
o Data collection tool
o Data collection procedure
o Data management and quality control
o Statistical data analysis
o Ethical consideration

**Response: Thank you for the suggestion, however this manuscript adopted the manuscript organization suggested by Plos Mental Health. This can be found in the journal’s website https://journals.plos.org/mentalhealth/s/submission-guidelines**
• Line 107: Sentence has grammar problem

**Response: The sentence redone for clarity as follows: These research assistants contacted the consenting HIV patients explaining the purpose and rational of the study**

5. Results

• Line 138: Values should be reported as % (n) such as 59.5% (n=122), while 55.1% (n=113) since it is the proportion that is reported and not numbers. And this should be affected across the manuscript.

**Response: The study is reporting both the numbers and proportions of the total hence they are better captured as they are currently started. General presentation is that one states the value and what proportion of the total it represents n (%)**

• Line 151: Sentence ……….”9) and the individual symptoms score (Table 2). Put table 2 in brackets and this should be the same across the manuscript where referenced in the text i.e. see also Line 156.

**Response: the use oof the brackets for figures and tables general depends on what sentence precedes the table or figure. If the sentence refers to the figure or a table as is the case for Table 2 in line 156, then the brackets are not used. Table/or figure are part of the sentence. *The median BAI score was 4 (IQR 1-9) and the individual symptoms score are listed in Table 2.* But when there is no connecting word between sentence and table or figure then one uses the brackets for figure or table example. ….. high viral loads were associated with increased anxiety levels (p = 0.026; Table 3).**

Compare

with
• Line 144: Sentence “………..of tuberculosis infection, and 35 (17.1%) had other comorbidities (Table 1)” is correct

**Response: The above response applies here as well. The use oof the brackets for figures and tables general depends on what sentence precedes the table or figure. If the sentence refers to the figure or a table as is the case for Table 2 in line 156, then the brackets are not used. Table/or figure are part of the sentence. *The median BAI score was 4 (IQR 1-9) and the individual symptoms score are listed in Table 2.* But when there is no connecting word between sentence and table or figure then one uses the brackets for figure or table example. ….. high viral loads were associated with increased anxiety levels (p = 0.026; Table 3).**
• Line 153: Positive correlation is mentioned but at what level and what was the strength of the correlation?

**Response: This was a chi-square test which is generally used to aid our understanding and interpretation of the connection between two categorical variables. The difference or connection is shown by a P value. The sentence has therefore been changed to read significant connection**

• Line 385: Sentence …..” Table 1: Baseline characteristics of the study population (n =205 patients)” term population should be replaced with study participants

**Response: This has been adopted as suggested**

• In table 1, 2 and 3: Values should be reported as % (n)

**Response: See, our response above. We repeat here as well. The study is reporting both the numbers and proportions of the total hence they are better captured as they are currently started. General presentation is that one states the value/number and what proportion of the total it represents n (%)**

• Tables should be presented as scientific tables

**Response. The tables are scientific tables. We think they show our results accurately**
• Figure has no title

**Response: Title of the figure inserted**

6. Discussion

• There is a lot of use of first and second person in the sentence constructions. I suggest that the author adopt the third person in the sentence construction.

**Response: The document edited to use third person as suggestion adopted**

• Demographic characteristics of the study participants are not discussed.

**Response: The manuscript concentrated the discussion around its objectives. Those variables (demographic/clinical/ laboratory results etc) that were found significant in multivariate models formed the main bedrock for the discussions**

• How do the present findings relate to the previous similar findings from other studies

**Response: The comparisons of these findings and others from previous studies has been discussed. The possible reasons for the current results have also been discussed**

• Line 168-169: Actual values should accompany the terms majority, moderate and severe based on the actual findings from the study.

**Response: We have included the percentages for these categories**

Then also tables and/ or figures should be cited.

**Response: Citing tables and figures in the discussion has been declined. The standard practice of the journal is that you don repeat results in the discussion section**

• Line 202: Sentence “……….among PLHIV. Consistent with this study Maier et al [9]…..,” et al should be written as “ et al. (9).

**Response: the suggestion adopted**

7. Conclusion

• It is missing in the document

**Response: The conclusion is provided in the last paragraph of discussion line 288……starting in summary**

Reviewer #4: This study describes a prevalence of anxiety among HIV patients in Kenya. correlations between anxiety and other factors are also described.

Abstract:

1. Line 31-32: were participants medically diagnosed with anxiety disorder? If this is from the BAI, address accordingly.

**Response: The Beck Anxiety Inventory (BAI) Questionnaire was used to assess anxiety levels, with a score of 8 or more indicating moderate to severe anxiety, as stated in the abstract."**

2. Line 33-34: this sentence can be made clearer. I am assuming participants do not have formal diagnosis of anxiety disorder

**Response: The issue to be made clearer has not been understood. It should be noted that the BAI anxiety scoring criteria has been tested and if responded to accurately would show the actual anxiety level**

**BAI was used to determine prevalence of anxiety disorder and it’s recognized to be very useful tool**
3. Line 35-37: this sentence can be made clearer

**Response: The comment noted but not clear what the issue to be addressed.**

4. Did duration on ART affect participant anxiety levels/severity?

**Response: The variables associated with anxiety are as listed in the abstract. All other variable found not significant have been listed in Table 4**

Introduction

1. 47: “mental disorders” is not needed

**Response: The term mental disorder as used in this sentence is used the show the overall name for all the mental issues**
2. 51,54: missing citation; please review all and make sure to cite

**Response: The citation added to the sentence**

3. 59-61: this sentence can be made clearer. What is meant by “interest in the public health context”
**Response: The sentence shows that although mental health is a universal health issue, it is yet to gain interest it deserve as a public health concern. Rephrased as follows “Although mental health is a universal human right according to World Health Organization (WHO), the mental health of PLHIV is often neglected and still need more attention and interest as a public health concern [13]”.**

Methods

1. 72: ART need to be fully spelled out as this is the first time it is mentioned in the article (not abstract)

**Response: ART written in full**

2. 80: capitalization

**Response: The first letters of Comprehensive Care Clinic (CCC) written in capital**

3. 86: What language was the study conducted in? was language an exclusionary criterion? Did participants fully informed?

**Response: Thank you for this question, The study was conducted in basic English to ensure all eligible participants could be enrolled. Any necessary clarifications were provided in simple terms to facilitate understanding.**

4. 107: typo, delete “the”

**Response: The section edited to remove typos**

Result
1. 146: missing punctation before “43”

**Response: Thank you for the observation, appropriate punctuation inserted**

2. 147-148: This sentence is incorrect as a score of 8 on the BAI indicates mild anxiety, see line 112.’

**Response: Thanks for the observation. This has been corrected as follows; The proportion of participants with a BAI score of 8 or more indicated mild to severe anxiety.**

3. 160-162: this could be made clearer; “households larger by one” and “smaller families” can be interpreted as the same thing. Additionally, did participants indicate whether they lived with families or friends or roommates?

**Response: Thank you for this observation. The household population clarified, Individuals from households larger than two members were 48% more likely to have anxiety compared to those from families with household comprising of one individual. The probing question was household population size (families only).**

Discussion

1. 167: this is not true as symptoms of anxiety were not evaluated (anxiety was measured using a questionnaire).

**Response: Thank you for the comment. The term 'symptoms' has been removed for accuracy. The revised sentence reads: 'This study found that 29.3% of PLHIV receiving ART had anxiety disorder as categorized by the BAI.**

2. Fix all the contractions. These are not allowed in research articles

**Response: Thank you for this comment. The document has been proof read to remove all the contractions used**

3. 185-188: Please explain this sentence, specifically on ‘biological’ differences between races and ethic **groups.**

**Response: Thank you for this observation. Several research show the potential role of genetic factors associated with anxiety disorder, which varies from race to race and ethnic background. Twin studies have estimated the heritability of anxiety disorder to be between 32% and 49% (Mather et al., 2016; Maron & Nutt, 2017). Genes for monoamine oxidase A *(MAOA)* and solute carrier family 6 member 4 *(SLC6A4)* are implicated in the pathogenesis of anxiety disorders (Tadic et al, 2003; You et al., 2005). We have added refences to qualify the sentence**

**References**

1. **Mather L. Blom V. Bergstrom G. Svedberg P. An underlying common factor, influenced by genetics and unique environment, explains the covariation between major depressive disorder, generalized anxiety disorder, and burnout: a Swedish twin study.*Twin Res Hum Genet.*2016. 19: 619-627**
2. **Tadic, A., Rujescu, D., Szegedi, A., Giegling, I., Singer, P., Möller, H. J., & Dahmen, N. (2003). Association of a MAOA gene variant with generalized anxiety disorder, but not with panic disorder or major depression. American Journal of Medical Genetics Part B: Neuropsychiatric Genetics, 117(1), 1-6.**
3. **You, Jin-Song, Sui-Yu Hu, Binglai Chen, and Hong-Gen Zhang. "Serotonin transporter and tryptophan hydroxylase gene polymorphisms in Chinese patients with generalized anxiety disorder." Psychiatric genetics 15, no. 1 (2005): 7-11.**
4. **Maron, E., & Nutt, D. (2017). Biological markers of generalized anxiety disorder. Dialogues in Clinical Neuroscience, 19(2), 147–158.**

4. 198: are all sub-Saharan African patriarchal? If this is a generalization, please address accordingly.

**Response: The sentence has been edited to limit to families, tribe, community and societies. Sub-Saharan Africa has been removed because the male headed societies are not only found in Africa alone but in different walks of life**

5. 200: “increased” implies causality. Please address and review such language throughout the paper

**Response: Increased replaced with associated with was associated with having anxiety disorder. The document edited to correct any miss implication**

6. 209: please include the specific finding related to household sizes. Also further discuss why household size was investigated in this study (social support and anxiety?)

**Response: Thank you for this comment; paragraph on household size and anxiety has been included in the discussion as follows: In the current study, participants from household with large population sizes (median IQR) were more likely to have anxiety disorder. Studies evaluating the role of family size and anxiety disorder are particularly rare among PLHIV. However, previous reports among the general population are conflicting, some show association between large family size and increased risk of anxiety disorder (McDonald et al., 2020) while others showing large family/household size being protective (Grinde and Tambs, 2016). Household population size is an indicator of both socioeconomic status as well as availability of social support (Hajebi et al., 2018). Anxiety disorders especially in large family sizes form poor and rural setting are thought to relate to limited resources rather than the family size (McDonald et al., 2022). In China anxiety disorder are shown to relate to poor family functioning and quality of life, as well as, lack of social support, more prevalent in small family sizes (Wang et al., 2016).**

**References
McDonald AJ, Hamilton HA, Elton-Marshall T, Nigatu YT, Jankowicz D, Bondy SJ, Wells S, Wickens CM. Household composition and anxiety symptoms during the COVID-19 pandemic: A population-based study. PLoS One. 2022 Nov 3;17(11):e0277243.**

**Grinde B, Tambs K. Effect of household size on mental problems in children: results from the Norwegian Mother and Child Cohort study. BMC Psychol. 2016 Jun 2;4(1):31.**

**Hajebi A, Motevalian SA, Rahimi-Movaghar A. *et al.* Major anxiety disorders in Iran: prevalence, sociodemographic correlates and service utilization. *BMC Psychiatry* 18, 261 (2018).**

**Wang J, Chen Y, Tan C, Zhao X. Family functioning, social support, and quality of life for patients with anxiety disorder. International Journal of Social Psychiatry. 2016;62(1):5-11.**

This manuscript can contribute to the literature on HIV and anxiety, specifically in Kenya. However, significant editing and further proofreading are needed.

**Response: We thank you for your comments which have improved this manuscript significantly**

7. PLOS authors have the option to publish the peer review history of their article ([what does this mean?](https://journals.plos.org/mentalhealth/s/editorial-and-peer-review-process#loc-peer-review-history)). If published, this will include your full peer review and any attached files.


**Do you want your identity to be public for this peer review?** If you choose “no”, your identity will remain anonymous but your review may still be made public.


For information about this choice, including consent withdrawal, please see our [Privacy Policy](https://www.plos.org/privacy-policy).

Reviewer #1: No

Reviewer #3: **Yes:** DR. Godfrey S. Bbosa

Reviewer #4: No

[NOTE: If reviewer comments were submitted as an attachment file, they will be attached to this email and accessible via the submission site. Please log into your account, locate the manuscript record, and check for the action link "View Attachments". If this link does not appear, there are no attachment files.]

While revising your submission, please upload your figure files to the Preflight Analysis and Conversion Engine (PACE) digital diagnostic tool, https://pacev2.apexcovantage.com/. PACE helps ensure that figures meet PLOS requirements. To use PACE, you must first register as a user. Registration is free. Then, login and navigate to the UPLOAD tab, where you will find detailed instructions on how to use the tool. If you encounter any issues or have any questions when using PACE, please email PLOS at figures@plos.org. Please note that Supporting Information files do not need this step.

*In compliance with data protection regulations, you may request that we remove your personal registration details at any time.*  [*(Remove my information/details)*](https://www.editorialmanager.com/pmen/login.asp?a=r)*. Please contact the publication office if you have any questions.*
